# An Unusual Autopsy Case of Artificial Conservation of a Corpse in a Domestic Setting

**DOI:** 10.7759/cureus.16382

**Published:** 2021-07-14

**Authors:** Mayank Gupta, Praveen K Tiwari

**Affiliations:** 1 Forensic Medicine, Institute of Medical Sciences, Banaras Hindu University, Varanasi, IND

**Keywords:** mummification, adipocere, preservation, clarified butter, turmeric powder

## Abstract

Decomposition, or the process by which a corpse turns into a skeleton due to the destruction of the soft tissue, is very complicated. Humankind has used various methods to inhibit and delay decomposition changes over centuries. It includes mummification, embalming, and preservation of the body under low temperatures. In this report, we present a criminal case involving the conservation of the body using a paste made from clarified butter and turmeric powder, with the body simultaneously kept in an air-conditioned room. The body acquired a stiff, leathery appearance consistent with mummification. The subjacent layers of the muscles had a soft consistency and specified adipocere changes. Adipocere changes were present in most internal organs. This case report involves a certified hospital death with no disagreement over the after-death interval or the cause of death during the postmortem examination. The postmortem changes resulted from a unique method of body preservation: a combination of lower temperature (by air conditioner) and the application of clarified butter mixed with turmeric powder.

## Introduction

Decomposition, or the process by which a corpse turns into a skeleton owing to the destruction of the soft tissue, is complicated. It involves cellular autolysis and tissue destruction. The release of endogenous compounds causes autolysis while tissue destruction is caused by either the releasing enzymes or the microorganisms present in the guts or outside [1]. Humankind has used various methods to delay or mitigate the decomposition changes. The reason for the preservation of the body may vary from cultural and religious beliefs, learning and research purposes, to criminal intention in rare cases.

Mummification was practiced in the ancient Egyptian culture for body preservation. The body was stripped of its internal organs, except for the heart, and dried with natron (a naturally-occurring salt). The ingredients of mummification balms were relatively economical fats and oils of plant and animal origin. Other, more expensive substances such as perfumes, spices, and herbs were added for their symbolic and preservation properties [2]. Embalming is yet another method of human cadaver preservation, especially for academic and research purposes. Embalming is the practice of injecting chemicals into a deceased person after death to keep the body from decaying. Formaldehyde, glutaraldehyde, phenol, glycerin, bronopol, ethanol, and glycol are among the common chemicals used in the embalming solution [3]. With the advances in technology, another form of anti-decomposition method has emerged, which involves storing the body in cold chambers at low temperatures, and it is a form of body preservation that significantly slows down the decomposition process [4].

In this report, we discuss a criminal case in which two sons applied an anti-decomposition method on their deceased mother's body and kept it for four months so that they could continue to collect her pension. They used an improvised method and applied a paste made from clarified butter and turmeric powder to the entire body on many occasions. Simultaneously, the corpse was held in an air-conditioned space in their house, causing the decomposition process to slow down.

## Case presentation

The deceased was a 70-year-old woman identified as Mrs. X, living at her residence in Varanasi, India, with her four sons. She died at 7 p.m. on January 13, 2018, at a local government hospital, from chronic liver disease/inflammatory bowel disease/hypothyroidism/high anionic gap metabolic acidosis with delirium, and disseminated intravascular coagulation/shock (death certificate was available).

Instead of performing the final rituals, the sons took the body home and kept it in an air-conditioned bedroom, now and then applying a paste made of clarified butter and turmeric. They aimed to conceal her death as long as possible in order to keep receiving her pension, for which the thumbprint impression of the deceased was required. After an extended power outage in the area, neighbors alerted the cops because of a foul odor coming from the home. During questioning by the police, the relatives claimed innocence and said that the mother was in a coma and that they had been providing her with medicines and milk daily. In May of 2018, the police sent the deceased's body to our department for a medicolegal autopsy to ascertain the cause of death and time since death. 

**Figure 1:**
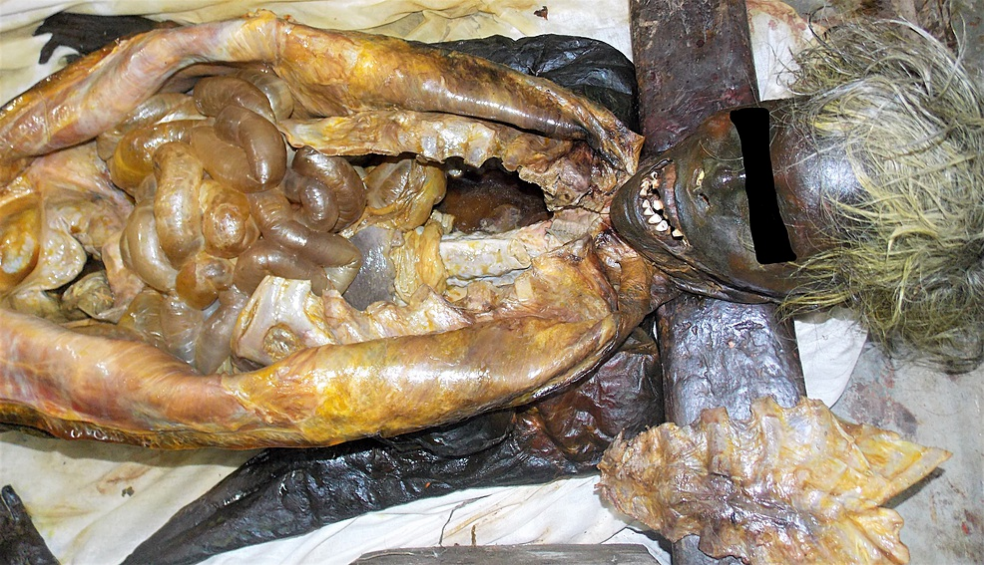
Mummified external body surface with adipocere changes present in internal organs and inner layers of the body trunk

**Figure 2:**
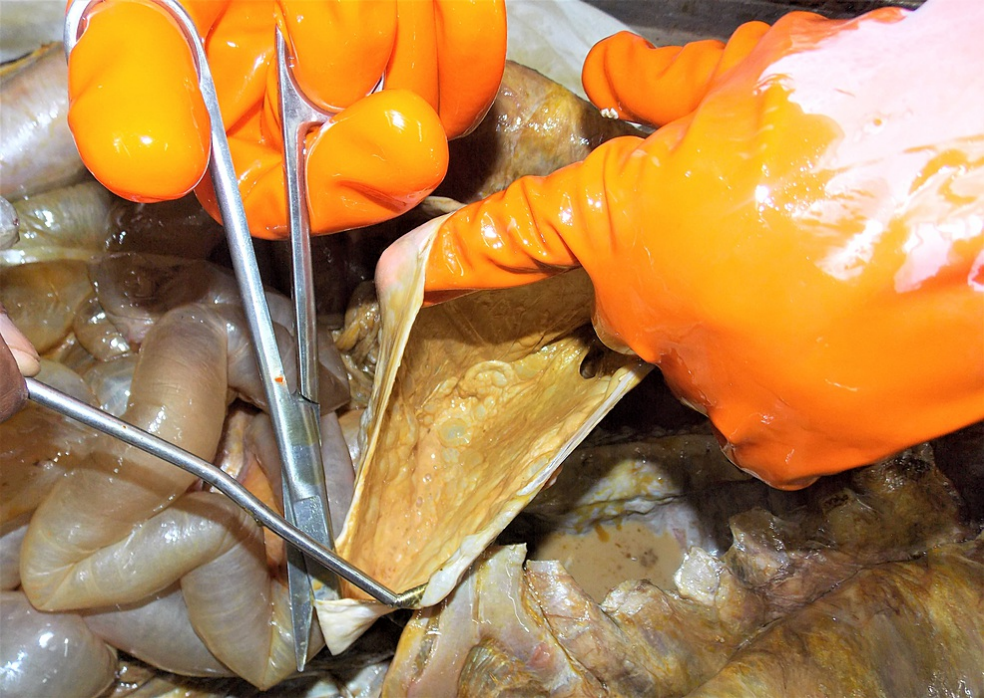
Stomach devoid of any content

External examination: the mummified body of an old-aged female found with hard leathery brownish-black body surfaces all over the body, except the back of body trunk where the body wall tissue is of soft adipose consistency, and sweetish disagreeable smell. The mouth was found open with a visible buccal cavity, the tongue was in a dried (mummified) state, and both eyes also appeared dried. The body surface had evidence of being applied an oily/greasy substance with yellow stains all over the body except for the back. On dissection: the external body wall tissue over the neck and front of the body trunk was found with a stiff, leathery appearance consistent with mummification. Meanwhile, the subjacent layers of muscle had a soft consistency and specified adipocere changes in their total thickness (body wall). Adipocere changes were present in all internal visceral and vital body organs; however, both kidneys and spleen were liquefied (Figure 1). The stomach was empty, which contradicted the claim that the deceased had been fed milk and medicines regularly (Figure 2). We did not find any evidence of antemortem or postmortem injuries on autopsy.

## Discussion

The practice of criminals destroying evidence, including the corpse, is well known, but criminals preserving the dead body is relatively uncommon in the literature. The 'Rhyl mummy' case, in which a woman was strangled and hidden in a cupboard while the culprit continued to claim her pension, is a famous one [5]. In April 2018 in Kolkata, a man was found to have preserved his mother's decomposing body in the freezer for three years to collect her pension, the motive being the same as in our case [6,7]. In our case, the process of preservation was performed by a combination of air conditioning and the application of a paste made from clarified butter and turmeric on the body. It led to an infrequent event in which the body's outer layers got mummified while the inner layers of the body wall and internal organs went into adipocere. Bhise et al. (2020) have described a case where they found a mixed picture of decomposition-mummification with adipocere formation in a body found hanging from a tree in the forest [8].

A body goes through several physicochemical changes after death. It gets influenced by factors inside and outside the corpse [9]. Saponification, or adipocere formation, is a natural preservation mechanism well known for centuries. Saponification needs favorable temperature for the production of the microbes and water, which can be exogenous or derived from the organism [1,10]. Adipocere formation occurs most often in fatty areas, including the cheeks, chin, belly, and buttocks [11,12].

Mummification is a natural or artificial preservation process that involves the dehydration and exsiccation (drying up) of tissues. It may be partial and coexist with other conservation or putrefaction processes. It extends more easily to the whole body than other processes, such as saponification [5]. Typical features associated with this process are dryness and brittle, torn skin on the bony prominences (cheeks, forehead, sides of the back, and hips). It is usually associated with the color brown but can coexist with white, green, or black zones due to fungus colonization [13,14]. Mummification occurs in dry, ventilated conditions [1,10]. More often than not, it occurs in warm places where the body loses fluids by evaporation: closed houses, attics, wardrobes and pantries, barns, or stairwells [1,15]. More widespread and complete mummification occurs in desert conditions; indeed, the ancient Egyptians practiced this preservation process, and they added spices and herbs on top of the heat [1,5,10].

Radanov et al. (1992) have presented a severe and unique case of natural brain mummification, which was unexpected given the softness of brain tissue and its fatty composition. The body had been buried for 40-50 years in a shallow depth in rocky terrain, exposed to sunshine, in a mass grave with 39 other corpses [16]. Dehydration before death facilitates the process of mummification. Indeed, there is an ancient Japanese tradition known as natural self-mummification, in which monks would gradually decrease their solid food consumption, then liquid intake, until they virtually desiccated themselves at the time of death. Three years after burial, their bodies were exhumed and discovered to be mummified, with no other external involvement [17].

Due to the usual long periods between death and the discovery of the body, the time required for mummification is not fully known. It may take a few weeks and may present with putrefactive alterations in the early stages, especially in the internal organs [1,5,18]. It is common to have slight adipocere in mummified bodies. Indeed, these two mechanisms are interconnected; body water used for fat hydrolysis also leads to tissue exsiccation [1,5]. In various parts of the body, partial mummification may coexist with other putrefactive modifications [13,14]. Makristathis et al. have found the same saponification constituents in mummies, including palmitic, oleic, and 10-hydroxystearic acid, among other substances, demonstrating that this relationship extends to the biochemical level [19].

## Conclusions

We presented a case of a certified hospital death of a woman on January 13, 2018, at 7:00 p.m. due to chronic liver disease/inflammatory bowel disease/hypothyroidism/high anionic gap metabolic acidosis with delirium, and disseminated intravascular coagulation/shock. There was no disagreement over the after-death interval or the cause of death during the postmortem examination. The sons tried to preserve the body with mal-intention. The postmortem modifications resulted from an unusual body preservation method: the combination of reduced temperature (by air conditioner) and the application of clarified butter and turmeric.
